# A Predictive Model for the Risk of Posterior Circulation Stroke in Patients with Intracranial Atherosclerosis Based on High Resolution MRI

**DOI:** 10.3390/diagnostics12040812

**Published:** 2022-03-15

**Authors:** Zhenxing Liu, Feiyang Zhong, Yu Xie, Xuanzhen Lu, Botong Hou, Keni Ouyang, Jiabin Fang, Meiyan Liao, Yumin Liu

**Affiliations:** 1 Department of Neurology, Zhongnan Hospital of Wuhan University, Wuhan 430071, China; 200432180216@whu.edu.cn (Z.L.); xieyuyy@163.com (Y.X.); 2014302180250@whu.edu.cn (B.H.); ouyangkeni2022@163.com (K.O.); fangjiabin2022@163.com (J.F.); 2 Department of Radiology, Zhongnan Hospital of Wuhan University, Wuhan 430071, China; feiyang.zhong@foxmail.com; 3 Department of Neurology, Wuhan Third Hospital, Wuhan 430060, China; lxz1232022@163.com

**Keywords:** vertebrobasilar artery, high resolution MRI, predictive model, nomogram

## Abstract

Intracranial vertebrobasilar atherosclerosis is the main cause of posterior circulation ischemic stroke. We aimed to construct a predictive model for the risk of posterior circulation ischemic stroke in patients with posterior circulation atherosclerosis based on high-resolution MRI (HR-MRI). A total of 208 consecutive patients with posterior circulation atherosclerosis confirmed by HR-MRI, from January 2020 to July 2021, were retrospectively assessed. They were assigned to the posterior circulation stroke (49 patients) and non-posterior circulation stroke group (159 patients) based on clinical presentation and diffusion-weighted imaging (DWI). Demographic data, risk factors of atherosclerosis, laboratory findings, and imaging characteristics were extracted from electronic health records. Plaque features were investigated by HR-MRI. Fifty-three clinical or imaging features were used to derive the model. Multivariable logistic regression analysis was employed to construct the prediction model. The nomogram was evaluated for calibration, differentiation, and clinical usefulness. Plaque enhancement, plaque irregular surface morphology, artery location of plaque, and dorsal quadrant of plaque location were significant predictors for posterior circulation stroke in patients with intracranial atherosclerosis. Subsequently, these variables were selected to establish a nomogram. The model showed good distinction (C-index 0.830, 95% CI 0.766-0.895). The calibration curve also showed excellent consistency between the prediction of the nomogram and the observed curve. Decision curve analysis further demonstrated that the nomogram conferred significantly high clinical net benefit. The nomogram calculated from plaque characteristics in HR-MRI may accurately predict the posterior circulation stroke occurrence and be of great help for stratification of stroke decision making.

## 1. Introduction

Stroke is the second leading cause of death in the world and the main cause of death in Asia [1]. Posterior circulation stroke accounts for about 20–30% of all ischemic stroke [2,3] and remains a significant cause of disability and mortality. Intracranial atherosclerotic disease (ICAD) is a major cause of ischemic stroke and is highly prevalent in China [4]. Furthermore, the risk of stroke recurrence is higher in patients with ICAD compared with any other subtype [5,6]. The underlying mechanisms for ICAD stroke are multifactorial and can, potentially, be inferred from plaque characteristics [7,8,9,10]. Compared with the efficacy of endovascular thrombectomy for anterior circulation large vessel occlusion in acute ischemic stroke, the result in posterior circulation was relatively disappointing [11,12], highlighting the significance for identifying the criminal lesions in early phase. Therefore, accurate risk assessment of intracranial plaque is helpful in guiding patient-specific treatment to prevent future stroke.

High-resolution magnetic resonance imaging (HR-MRI) is a novel imaging assessment technology which has emerged in recent years, and it has been widely applied in the evaluation for plaque characteristics [13]. Compared with digital subtraction angiography (DSA), HR-MRI has advantages, including high resolution and visualization of the vascular wall structure, as well as non- invasiveness. It has been regarded as a reliable method for evaluating plaque characteristics [13]. HR-MRI could be applied in assessing the plaque location, burden, enhancement ratio, size, length, and area, as well as the remodeling index both qualitatively and quantitatively [10,13,14,15]. Several studies have shown that plaque features can provide additional value to the measured degree of stenosis in differentiating stroke from asymptomatic patients. In these studies, intracranial atherosclerotic plaque (ICAP) features based on HR-MRI, including positive remodeling [16], diffuse distribution [17], intraplaque hemorrhage [8] and strong enhancement [18], have been identified as markers for stroke risk factors. In particular, enhancement is thought to be of great importance for assessment of plaque vulnerability [19,20]. However, most of the research into the association between stroke and intracranial atherosclerosis plaque characteristics focus on the anterior circulation [20,21]; limited investigations were related to the posterior circulation. Therefore, the findings might be questionable in the risk assessment of patients with intracranial vertebrobasilar atherosclerosis.

Therefore, a scoring system accurately predicting posterior ICAS risk is needed. Nomograms have been gradually applied in medical research and clinical practice. They are used for visualization of multiple indicators to diagnose or predict disease onset or progression, making prognostic model results easier to read [22]. To the best of our knowledge, few risk models based on arterial plaque characteristics by HR-MRI have been developed in predicting the risk of posterior circulation ischemic stroke. In this study we developed an effective risk nomogram aiming to investigate the relationship between plaque features and posterior circulation stroke occurrence. Our nomogram will facilitate for risk stratification and better use of medical measures and will finally reduce the poor outcomes of patients with posterior ICAS.

## 2. Materials and Methods

### 2.1. Study Population

We retrospectively analyzed patients who underwent HR-MRI from January 2020 to July 2021 in Zhongnan Hospital of Wuhan University. The patients who presented with posterior circulation ischemic symptoms and had intracranial atherosclerotic plaques in vertebrobasilar artery identified on HR-MRI were enrolled in this study. The exclusion criteria included: (1) patients with co-existence of 50% ipsilateral extracranial vertebral artery stenosis; (2) patients with intracranial arterial disease caused by non-atherosclerotic entities according to the HR-MRI findings and clinical laboratory examination (such as aneurysm, moyamoya disease, vasculitis, arterial dissection, reversible cerebral vasoconstriction syndrome, or dysplasia); (3) patients with no more than one risk factor for atherosclerosis, such as hypertension, diabetes, dyslipidemia, overweight and obesity, or smoking; (4) patients with risk factors for cardioembolic strokes, such as atrial fibrillation, acute myocardial infarction, patent foramen ovale, valvular heart disease, or cardiomyopathy; and (5) poor HR-MRI imaging. All the information was anonymous, and confidentiality of information was assured.

We collected the general data of patients who met the inclusion criteria, including sex, age, body mass index (BMI), past medical history (hypertension, hyperlipidemia, coronary heart disease [CHD], stroke history, etc.), and laboratory indexes (red blood cell count, white blood cell count, platelet count, neutrophils count, lymphocytes count, alanine transaminase [ALT], aspartate aminotransferase [AST], albumin, blood urea nitrogen, serum creatinine, uric acid, blood glucose, total cholesterol, triglyceride, low density lipoprotein cholesterol [LDL-C], high-density lipoprotein cholesterol [HDL-C], lipoprotein a, high homocysteine, etc.), smoking history, clinical symptoms, and imaging data. Furthermore, neutrophil-lymphocyte ratio and platelet-lymphocyte ratio were calculated according to the full blood counts on admission.

Patients were assigned to the stroke group when they had symptoms of neurological impairment caused by posterior circulation ischemia, and ischemia foci confirmed by diffusion-weighted imaging (DWI) was consistent with clinical symptoms. Transient ischemic attack (TIA) patients manifested as posterior circulation symptoms and signs, such as vertigo combined with partial limb numbness and weakness, blurred vision, dysarthria etc., were also assigned to the case group. Patients without central nervous system function deficit and MRI that did not identify a newly infarct site in the posterior circulation area, were assigned to the non-stroke group.

### 2.2. HR-MRI and Vessel Wall Imaging Assessment

HR-MRI images were obtained with a Siemens 3T Prisma scanner using a 64-channel head coil. Imaging parameters were set according to the clinical practice. Imaging data were reviewed on the picture archiving and communication system (PACS) by two experienced neuroradiologists who were blinded to the clinical characteristics. The reproducibility of imaging feature quantification was calculated by comparing the results from the two reviewers in 30 random samples. Cases that have disagreements were reviewed by a higher-level neuroradiologist to make the final judgment.

Plaque enhancement (PE) was classified using a previously established grading scheme [23]: no enhancement, ≤the degree of enhancement of arterial walls without plaque in the same vessel; mild enhancement, between the degree of enhancement of the normal vessel wall and the degree of enhancement of the pituitary infundibulum; and marked enhancement, ≥the degree of enhancement of the pituitary infundibulum. At the narrowest vessel wall, plaque distribution patterns were identified. The anatomical locations of plaques were described as ventral, dorsal, left, and right quadrants. Plaque that was distributed across ≤2 quadrants of the lumen perimeter was defined as focal and that involving ≥3 quadrants was defined as diffuse. Plaque surface morphologies were divided into regular and irregular types.

The measurements of the vessel area (VA) and lumen area (LA) were performed on cross-sectional T1-weighted images at the maximal lumen narrowing (MLN) and reference sites. The reference site was selected based on the WASID method [24]. The remodeling index (RI) was defined as, [VA _MLN_/VA _reference_]; RI ≥ 1.05 was defined as positive remodeling, RI ≤ 0.95 as negative remodeling, and RI between 0.95 and 1.05 as intermediate remodeling. Wall area (WA) was measured using VA–LA, and plaque burden was measured on the maximal stenosis site and was defined as (WA/VA) × 100% [25,26]. A standardized method was applied to measure luminal stenosis [24]. Percent luminal area stenosis (% stenosis area) was calculated as % stenosis area = (1 − LA_MLN_/LA_reference_) × 100%.

On the reconstructed post-contrast images, at the site of the most stenotic lesion or the most apparent wall thickening, maximum plaque length and maximum wall thickness were measured, respectively. The ratio of maximum plaque length to maximum wall thickness was calculated.

The geometry of vertebrobasilar artery was qualitatively categorized into four basic geometric configurations: Walking, Tuning Fork, Lambda, and No Confuence [27].

### 2.3. Statistical Analysis

R version 4.0.5 (31 March 2021) (http://www.Rproject.org) was employed for data analysis. Continuous variables were described as mean ± standard deviation (SD) or median [interquartile range] (IQR), for normal or non-normal distributions, and categorical variables were expressed as frequency and percentage. Student’s t test or Wilcoxon rank sum test was used for the comparison of continuous variables, and the chi-square or Fisher’s exact test was used for the comparison of categorical variables, as appropriate. All tests with two-sided *p* < 0.05 was considered statistically significant. The R package “tableone” was used in these analyses. Reproducibility of plaque features was evaluated by intraclass coefficient (ICC) using a two-way random model with absolute measurements. The R package“irr”was used in the ICC algorithm.

Firstly, we performed least absolute shrinkage and selection operator (LASSO) logistic regression algorithmto retain the most predictive features. Ten-fold cross-validation was used to estimate LASSO hyperparameters. In addition, we performed a univariate logistic regression to identify the parameters selected, in which those with a *p* < 0.05 were then incorporated in a multivariate logistic regression, followed by backward stepwise selection method. Furthermore, we constructed predictive models using logistic regression, decision tree, and support vector machine (SVM) via the R packages “rpart”, “rpart.plot”, “randomforest”, “e1071”and “caret” to compare the advantage of different models. The Delong test was carried out for assessing differences in AUCs in various models. By combining the final significant predictors, we established a nomogram to predict the risk of the posterior circulation ischemic stroke. Nomogram performance was assessed by a calibration plot to indicate internal calibration, the Hosmer–Lemeshow test to evaluate the goodness of fit, and the area under the curve (AUC) to measure discriminative ability. Decision curve analysis (DCA) was conducted to evaluate the clinical utility of the nomogram by quantifying net benefits against a range of threshold probabilities. The R packages “glmnet”, “rms”, “pROC”, “dca.R”, and “ResourceSelection” were used in these analyses.

## 3. Results

### 3.1. Clinicopathologic Characteristics of Enrolled Patients with Posterior ICAS

**Figure 1:**
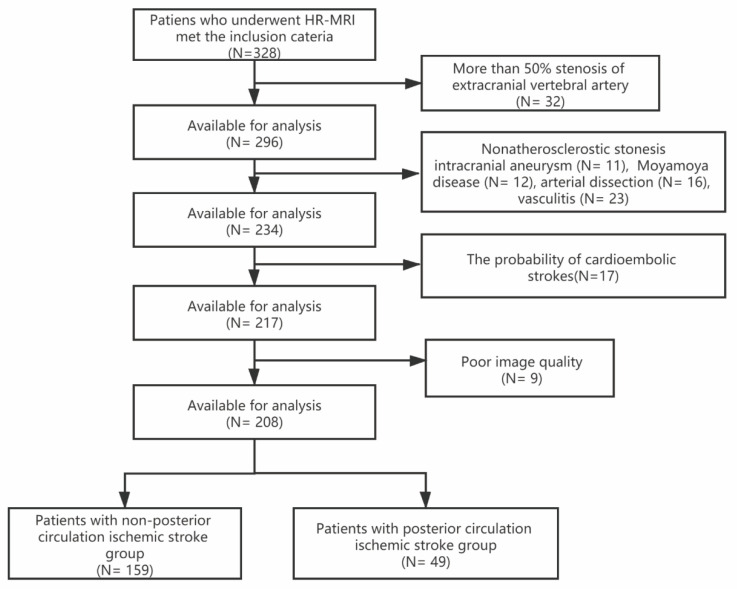
The flow chart for the inclusion of patients. HR-MRI, high-resolution magnetic resonance imaging.

The flow chart is shown in Figure 1. A total of 328 patients were included originally in the study. Of these, 120 patients were excluded due to >50% stenosis of extracranial vertebral artery (*n* = 32), intracranial aneurysm (*n* = 11), Moyamoya disease (*n* = 12), dissection (*n* = 16), vasculitis (*n* = 23), the probability of cardioembolic strokes (*n* = 17), and poor image quality (*n* = 9). Finally, a total of 208 patients (median age: 61 years; mean ± SD 60.2 ± 10.2, range 27–83) were enrolled in this study. A total of 152 (73.1%) were male patients; there were 159 patients in non-posterior circulation ischemic stroke (non-PCIS) group and 49 patients in PCIS group. The clinical and intracranial plaque characteristics and comparison between stroke and non-stroke patients were summarized in Table 1.

### 3.2. Comparison of Clinical and Intracranial Plaque Characteristics between Non-PCIS Patients and PCIS Patients

There were no significant differences in age, gender, or other comorbidities between these two groups. Patients in the stroke group had higher level of BMI, SBP, DBP, and MAP than those in the non-stroke group. Laboratory indicators which showed significant differences between two groups were: WBC, RBC, CHOL, TG, HDL, and LDL. As for artery plaque characteristics, compared with patients in the non-stroke group, stroke group patients presented more severe degree of luminal stenosis (0.32 [0.10, 0.56] vs. 0.56 [0.34, 0.88], *p* < 0.001), more severe degree of plaque burden (0.78 [0.69, 0.85] vs. 0.87 [0.79, 0.96], *p* < 0.001), and higher rates in maximum wall thickness (1.37 [1.02,1.91] vs. 1.58 [1.28, 2.11], *p* = 0.017). In addition, there were significant differences between the two groups in plaque distribution patterns, dorsal and right quadrant of plaque location, plaque enhancement degrees, plaque surface morphology, and the artery of plaque location. No significance was found in the type of artery remodeling, maximum plaque length, and geometry of the vertebrobasilar between the two groups. Markedly, higher proportion of marked plaque enhancement (35, 59.3%), irregular plaque surface morphology (51, 86.4%), and plaque location in basal artery (29, 49.2%) were observed in the stroke group.

### 3.3. Identification of Significant Predictors for the Risk of Posterior Circulation Ischemic Stroke

**Figure 2:**
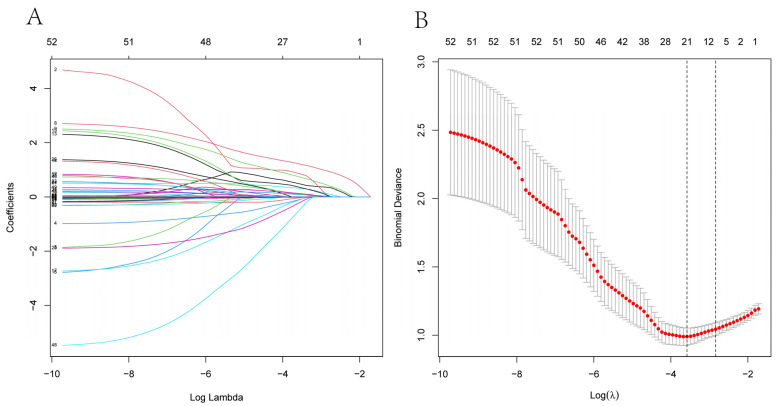
The result of LASSO model: (**A**) LASSO coefficient profiles of the candidate predictors. (**B**) The features with nonzero coefficients are shown in the model. LASSO, least absolute shrinkage and selection operator.

A total of 53 potential predictors were enrolled in LASSO regression, as shown in the Figure 2, and 21 candidate predictors with nonzero coefficients were selected, including the degree of luminal stenosis, plaque burden, the type of artery remodeling, the degree of plaque enhancement, the plaque surface morphology, the artery of plaque location, the dorsal and right quadrant of plaque location, the gender, the age, SBP, the history of dyslipidemia, the stroke history, WBC, UA, CHOL, LDL, HDL, LDa, fibrinogen, and d-dimer.

The above selected variables were analyzed by univariate logistic analysis, and the characteristics with a *p*-value < 0.05 were further introduced into a multivariate logistic regression. The results demonstrated that the degree of plaque enhancement (Mild vs. No: odds ratio [OR] = 5.47, 95%confidence interval [CI]:0.86~108.84, *p* = 0.131; Marked vs. No: OR = 28.93, 95% CI = 4.40~588.77, *p* = 0.003), plaque surface morphology (irregular vs. regular: OR = 3.26, CI = 1.34~8.75, *p* = 0.013), the artery location of plaque (left vertebral artery vs. right vertebral artery: OR = 0.76, CI = 0.29~1.97, *p* = 0.569; basal artery vs. right vertebral artery: OR = 3.14 CI = 1.24~8.29, *p* = 0.018), dorsal quadrant of plaque location (OR = 2.88, CI = 1.24~7.24, *p* = 0.018), systolic blood pressure (OR = 1.02, CI = 1.00~1.04, *p* = 0.044), and white blood cell (OR = 1.21, CI = 1.01~1.48, *p* = 0.048) were significant predictors for posterior circulation stroke event. As the WBC and the level of SBP are not a relative stable value due to the various conditions in the clinical practice, inclusion of them might have caused the predicted model unstable. Moreover, the subsequent analysis confirmed that the inclusion and exclusion of the two variables in the model did not make much more significant difference as shown in the Supplement Figure S1. Finally, the remaining four variables based on imaging features were included in the final model. Then, we used logistic regression, support vector machine (SVM), and a decision tree to construct different predictive models. The receiver operating characteristic (ROC) curve indicated that logistic regression was better than SVM and the decision tree (Supplementary Figure S2). Logistic regression model, therefore, was used for further analysis due to its better performance.

### 3.4. Construction and Validation of the Predictive Nomogram

**Figure 3:**
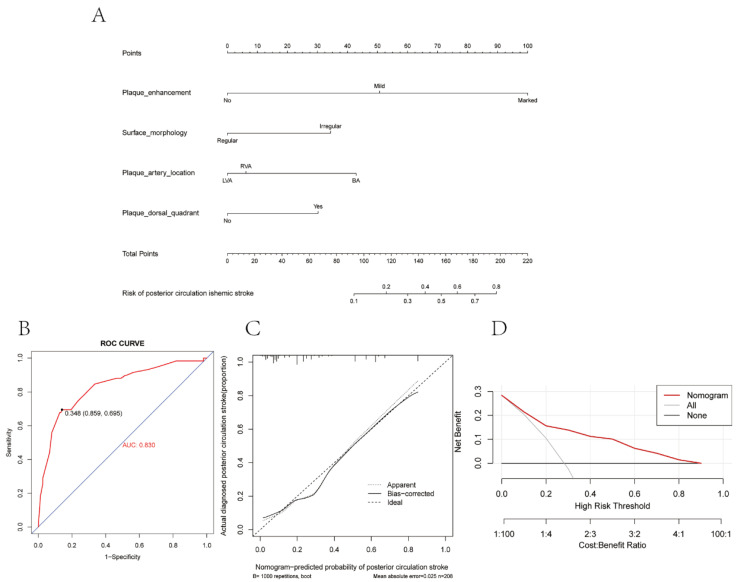
Construction and validation of the predictive nomogram for the risk of posterior circulation stroke in patients with ICAS. (**A**) Development of the nomogram to predict the risk of posterior circulation stroke in patients with ICAS. (**B**) Receiver operating characteristic (ROC) curves of the nomogram. (**C**) Calibration curve of the nomogram. (**D**) Decision curve analysis in the cohort. The y-axis represents net benefits, calculated by subtracting the relative harms (false positives) from the benefits (true positives). The x-axis measures the threshold probability. VB, basilar artery; LVA, left vertebral artery; RVA, right vertebral artery.

Based on the significant predictors above, we established a predictive nomogram for the stroke risk of patients with posterior circulation intracranial atherosclerosis. (Figure 3A). The four predictors were assigned a score ranging from 0 to 100 on a point scale. After adding the scores of all variables to “Total Points,” the corresponding value is the probability of risk of posterior circulation ischemic stroke. Regarding the clinical application of this nomogram, we assumed that if a patient’s degree of plaque enhancement was marked, the morphology of plaque surface was irregular, the location of plaque was in basal artery, and the location of plaque was not in dorsal quadrant, then the corresponding score of the risk was 100 + 34 + 43 + 0, a total of 177 points; therefore, so the corresponding risk of post circulation ischemic stroke of this patient was between 0.6 and 0.7, and this patient was in a relatively moderate-high risk of the stroke.

Based on the maximum Youden index, the optimal cutoff value of the nomogram predicted probability was set as 0.348, and the corresponding total points was 138.2. At this cutoff, the sensitivity, specificity, positive predictive value, and negative predictive value, when used in differentiating the presence from absence of risk of posterior circulation stroke, were 69.5%, 85.9%, 66.1%, and 87.7%, respectively. The lesion would be regarded as higher probability of stroke when the total prediction probability is beyond the cutoff point. Figure 3C shows the calibration curves for the nomogram model. The calibration curve and a significant Hosmer–Lemeshow test statistic (X-squared = 4.726, *p*-value = 0.787) showed good agreement between the predicted model and actual observation in the study, along with the C-index (0.830, 95% CI 0.766~0.895) indicating that the nomogram is a good discriminant tool. Furthermore, we performed a 5-fold 100-times cross validation, the result of C-index was 0.795 (95%CI, 0.788–0.802). Decision curve analysis (DCA) was used to assess the clinical utility of the nomogram by quantifying the probabilities of net benefits at a threshold from 0.0 to 1.0. DCA results showed that using this nomogram to predict the risk of posterior circulation stroke had more benefits than the measures that treat all patients or treat none of patients (Figure 3D).

### 3.5. Reproducibility of Plaque Feature Measurements

The ICCs of the plaque features for the two reviewers in measuring maximum plaque length, plaque enhancement, plaque surface morphology, artery location of plaque, and dorsal quadrant of plaque location were 0.853, 0.928, 0.869, 0.875, and 0.914, respectively

## 4. Discussion

With the advent of HRMRI for its great advantage on visualizing intracranial vessel walls, intracranial plaque features and arterial remodeling have become a new research focus. It provides us with deep insight into stratifying vulnerability of atherosclerotic plaques and identifying stroke etiology, as well as predicting individual risk of ischemic stroke. The role of the plaque characteristics attributing to stroke is still a controversy; however, plaque enhancement, positive remodeling and surface irregularity have emerged as strong imaging biomarkers of symptomatic plaque. The nomogram is a way of visualizing logistic regression and can be directly and easily used in the clinical study. In the present study, we performed a nomogram based on imaging features to clarify the association between plaque characteristics and stroke occurrence.

In our study, degree of plaque enhancement, morphology of plaque surface, artery of plaque location, and dorsal quadrant of plaque location were finally selected as predictors for posterior circulation stroke in patients with symptomatic intracranial atherosclerosis. Based on these predictors, we developed an individualized nomogram, which had a good identification effect (AUC = 0.830). Moreover, the calibration curve demonstrated excellent consistency between the prediction of the nomogram and the observed curve, while DCA further showed that the nomogram conferred significantly high clinical net benefits. Collectively, these findings suggested that the nomogram was of significant value for accurate assessment of the incidence of posterior circulation stroke in patients with symptomatic intracranial atherosclerosis and, thus, had the potential to provide support for decision-making as an early prevention method for posterior circulation stroke.

**Figure 4:**
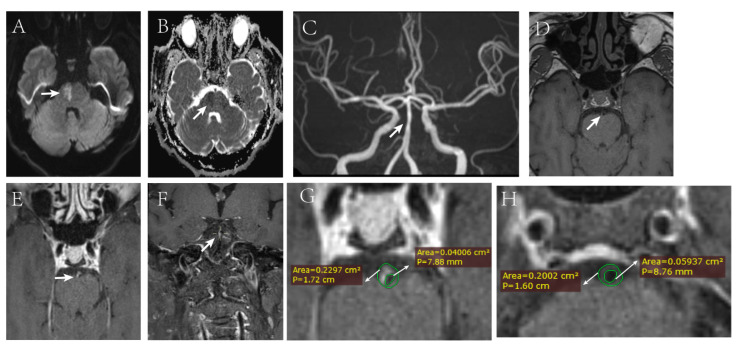
A 57-year-old male patient presented with dizziness accompanied by left limb weakness for 1 day. DWI and ADC showed limited diffusion in the right brainstem (**A**,**B**). MRA showed there was a mild stenosis in basilar artery (**C**). The 3D-T1-SPACE images perpendicular to the long axis of the vessel wall shows the irregular plaque in the ventral and right side of the vessel wall before enhancement (**D**), and the plaque is obviously strengthened after enhancement (**E**,**F**). The vessel area (VA) and lumen area (LA) were measured at the maximal lumen narrowing (MLN) and reference sites (**G**,**H**). The remodeling index (RI) was calculated as 0.2297/0.2002(1.147), the result was ≥ 1.05, so it was regarded as positive remodeling. Wall area (WA) was measured using VA–LA, WA_MLN_ = 0.1897 (0.2297 − 0.040), WAreference = 0.1408 (0.2002 − 0.0594). Plaque burden was calculated as 0.1897/0.2297 (0.826), and percent luminal area stenosis was calculated as (1 − 0.040/0.059) × 100% (32.2%). DWI, diffusion-weighted image; ADC, apparent diffusion coefficient.

Of note, our results were consistent with previous studies which showed that plaque enhancement was a strong predictive imaging biomarkers for stroke occurrence and recurrence [19,23,28,29]. The marked plaque enhancement played a predominate role in predicting stroke event. Moreover, the degree of plaque enhancement could also be used as a risk factor for predicting post-operative perforator stroke in patients with severe vertebrobasilar artery stenosis [29]. The mechanism of intracranial atherosclerosis plaque enhancement after contrast agent injection may be related to neovascularization, inflammation, and endothelial dysfunction in atherosclerotic plaque leading to leakage of gadolinium [30,31,32]. In addition, Yang et al. [33] found that poor posterior collateral circulation may be associated with high risk of plaque enhancement in patients with severe symptomatic intracranial vertebrobasilar atherosclerosis. The potential reason was that the pressure difference around the plaque may result in high plaque wall stress, increased endothelial permeability and, thus, plaque enhancement. In all, as it is shown in Figure 4, the phenomenon that plaque enhancement as a strong predictor of stroke event is critical for early management decisions in clinic practice and the due measures to prevent the occurrence of adverse events should be taken in time.

Plaque surface irregularity was also an important predictor of stroke in our final model. Symptomatic plaques seem to have a more irregular surface [34,35]. Fang et al. [36] found that plaque surface irregularity was regarded as part of vulnerable plaque characteristics, predicting artery-to-artery embolic infarction. Plaque rupture of the fibrous cap, manifesting as plaque surface irregularity, has been histologically validated in carotid endarterectomy specimens [37]. The alike explanation might apply for the result in intracranial atherosclerotic plaque. Furthermore, it is assumed that compared with regular-surface plaque, local hemodynamic changes around the irregular-surface plaque are more prone to promote thrombosis and the effect of long-term shear force induces vascular remodeling, and eventually lead to stroke events.

The posterior circulation has more variable hemodynamic changes than the anterior circulation due to the diverse tortuosity of vertebrobasilar arteries. Therefore, we included different shapes of vertebrobasilar artery geometry [27] to investigate their associations with the stroke occurrence in the study; however, no statistically significant conclusions were found. In addition, we found that atherosclerotic plaque has a different risk profile in different artery location, as shown in the nomogram. The basilar artery has more perforator openings, which may account for the higher risk of stroke. Interestingly, overall, we found more plaque in the left vertebral artery compared with right vertebral artery, 83 (39.9%) vs. 64 (30.8%), which is consistent with the study of Cai et al. [38]. More culprit lesions, however, were found lying in the right vertebral artery in the case set (right vs. left, 16 [27.1%] vs. 14 [23.7%], respectively). It should be viewed with caution due to the small sample size of the case set in our study and, thus, needs to be validated in a prospective study with a larger sample size.

In our study, we found that the atherosclerotic plaque in the dorsal quadrant in vertebrobasilar artery had higher risk of stroke than that in other quadrants. The result of distribution of culprit plaque in basilar artery (BA) in previous studies were not consistent. Studies conducted by Chen et al. [39] and Wang et al. [9] found symptomatic atherosclerotic plaques predominantly located in the ventral or lateral wall of BA where most perforators originate, while Yu et al. [10] observed the plaques caused symptomatic pontine infarctions were mostly distributed in the dorsal and bilateral walls of the basilar artery in patients with mild basal arterial stenosis. The cause of the discrepancy above might be due to the inconsistency of degree of basilar artery stenosis when inclusion of the studies performed. However, few similar investigations have ever been performed in the overall intracranial vertebrobasilar artery system. Whether the dorsal distribution of plaque in intracranial vertebrobasilar artery represents a higher risk of stroke requires further investigation.

Arterial stenosis has long been a subject of great concern for many researchers, and high grade of artery stenosis caused by atherosclerotic plaque is significantly related to the occurrence and recurrence of stroke [40]. However, a growing number of studies have shown that other features of plaque, such as plaque enhancement [20] and plaque burden [25], are more predictive than stenosis rate. Recently, Wang et al. [41] performed a systematic review on HR-MRI in which found even with zero or mild stenosis, intracranial high-risk plaques still have great associations with ischemic stroke and unfavorable outcome. In our study, univariate analysis suggested that a more severe stenosis rate was associated with a higher risk of stroke, whereas in multivariate regression, it was not significant after adjusting the effects of other plaque feature covariates, which suggested that other imaging features had higher predictive value than stenosis rate. In addition, we found that the AUC value of nomogram was higher than that of the stenosis rate, which is basically consistent with the results of other studies [20,25]. Furthermore, when we included the stenosis rate in the final nomogram model, no significant incremental value was found (Supplementary Figure S3). Thus, in clinical practice, more attention should also be placed on other high risk plaque characteristics besides the degree of artery stenosis.

Not consistent with other studies [21,42], no significant difference was found in positive remodeling between the two groups in the current study. This might be explained by the inclusion criteria with no strict control on the degree of lumen stenosis. In the early stage of atherosclerosis the positive remodeling as a method of adaptive compensation of atherosclerotic plaques makes it possible for the lumen area to be absent of sever luminal narrowing, even no stenosis occurs [26]; while lumen stenosis of varying degrees is always accompanied with large plaque volume, a thin fibrous cap, irregular plaque surface, and diffuse distribution were invariably observed in the positive remodeling pattern in the advanced stage of atherosclerosis [7], whereas the combination of the above factors represents a considerable risk of ischemic stroke. Hence, further comparisons regarding the remodeling patterns based on different percent of stenosis are needed in the future analysis, in which the more precise answer might have attained.

To our best knowledge, this is the first nomogram analysis for predicting the incidence of posterior circulation stroke in patients with symptomatic intracranial atherosclerosis. The greatest advantage is that this practical quantitative prediction tool is easily used and popularized, because only four parameters based on HR-MRI are needed, which are easily accessible in clinical practice. However, there are also some limitations in our study. First, as a retrospective study, it possibly has inclusion bias in the process; not all the patients with intracranial atherosclerosis were capable of attending this program in time due to various reasons. Second, we only included Chinese people, mainly in Hubei Province, which may limit generalizability of our study result to other region and ethnic people. Thus, external validation from other countries and races is necessary to confirm the predictive value of the nomogram. Finally, considering the plaque enhancement accounts for larger proportion of the nomogram, further study is needed into how the enhancing plaque varies over time. Moreover, it is hard to ascertain what the plaque enhancement of intracranial atheroma actually stands for in vivo since we could not perform pathologic analysis of the enhancing plaque. As for other plaque characteristics in the nomogram model, further investigations of their effect upon the stroke event are also needed in the future.

## 5. Conclusions

In conclusion, we demonstrated that plaque enhancement, plaque surface morphology, artery location of plaque, and dorsal quadrant of plaque location are significant predictors of posterior circulation stroke in patients with symptomatic intracranial atherosclerosis. By combining these predictive factors, a risk nomogram was established to predict the incidence of posterior circulation stroke in patients with intracranial atherosclerosis. The application of nomogram in a clinical setting could optimally assist in reducing the occurrence of posterior circulation stroke events and avoiding poor outcomes as possible. In addition, larger and multicenter studies are needed to verify the consistency of our findings and the external generalizability of the model in the future.

## Figures and Tables

**Table 1:** Clinical and intracranial plaque characteristics of study population and comparison between stroke and non-stroke patients.

Variables	All Patients (*n* = 208)	Non-Stroke Group (*n* = 159)	Stroke Group (*n* = 49)	*p*-Value
Age (year)	61.00 [54.00, 68.00]	62.00 [54.00, 68.00]	59.00 [53.00, 66.50]	0.174
Gender				0.575
Female (%)	56 (26.9)	38 (25.5)	18 (30.5)	
Male (%)	152 (73.1)	111 (74.5)	41 (69.5)	
BMI (kg/m^2^)	25.39 [23.25, 27.14]	24.98 [23.01, 26.45]	25.95 [24.38, 27.59]	0.012
SBP (mmHg)	146.0 [131.00, 157.25]	145.00 [130.00, 153.00]	151.00 [138.50, 168.00]	0.003
DBP (mmHg)	84.00 [75.00, 95.00]	81.00 [73.00, 93.00]	87.00 [80.00, 98.00]	0.009
MAP (mmHg)	104.00 [94.67, 115.67]	102.67 [93.33, 113.33]	107.67 [100.67, 121.17]	0.003
Comorbidities, *n* (%)				
Hypertension	174 (83.7)	121 (81.2)	53 (89.8)	0.191
Diabetes	79 (38.0)	56 (37.6)	23 (39.0)	0.977
Dyslipidemia	66 (31.7)	53 (35.6)	13 (22.0)	0.084
Coronary heart disease	28 (13.5)	18 (12.1)	10 (16.9)	0.483
Previous stroke history	64 (30.8)	50 (33.6)	14 (23.7)	0.223
Smoking	92 (44.2)	67 (45.0)	25 (42.4)	0.854
Plaque characteristics				
Luminal stenosis (%)	0.41 [0.14, 0.65]	0.32 [0.10, 0.56]	0.56 [0.34, 0.88]	<0.001
Plaque burden (%)	0.80 [0.71, 0.88]	0.78 [0.69, 0.85]	0.87 [0.79, 0.96]	<0.001
Remodeling index (%)	1.10 [0.98, 1.21]	1.09 [0.97, 1.23]	1.10 [0.99, 1.17]	0.729
The type of remodeling (%)				0.655
Negative remodeling	40 (19.2)	30 (20.1)	10 (16.9)	
Intermediate remodeling	35 (16.8)	23 (15.4)	12 (20.3)	
Positive remodeling;	133 (63.9)	96 (64.4)	37 (62.7)	
Distribution patterns (%)				0.006
Diffuse	126 (60.6)	81 (54.4)	45 (76.3)	
Focal	82 (39.4)	68 (45.6)	14 (23.7)	
Quadrant Location (%)				
Ventral	151 (72.6)	103 (69.1)	48 (81.4)	0.107
Dorsal	139 (66.8)	92 (61.7)	47 (79.7)	0.021
Left	166 (79.8)	114 (76.5)	52 (88.1)	0.091
Right	144 (69.2)	96 (64.4)	48 (81.4)	0.027
Maximum wall thickness (mm)	1.46 [1.09, 2.01]	1.37 [1.02, 1.91]	1.58 [1.28, 2.11]	0.017
Maximum plaque length (mm)	5.56 [3.77, 10.57]	5.47 [3.99, 9.88]	6.45 [3.32, 11.30]	0.802
Ratio of maximum length to thickness	3.87 [2.50, 6.56]	4.17 [2.56, 6.68]	3.45 [2.26, 6.32]	0.18
Plaque enhancement (%)				<0.001 *
NO enhancement	15 (7.2)	14 (9.4)	1 (1.7)	
Mild enhancement	132 (63.5)	109 (73.2)	23 (39.0)	
Marked enhancement	61 (29.3)	26 (17.4)	35 (59.3)	
Plaque surface (%)				<0.001
Regular	73 (35.1)	65 (43.6)	8 (13.6)	
Irregular	135 (64.9)	84 (56.4)	51 (86.4)	
Geometry of the vertebrobasilar (%)				0.669
Walking	55 (26.4)	41 (27.5)	14 (23.7)	
Tuning Fork	59 (28.4)	44 (29.5)	15 (25.4)	
Lambda	61 (29.3)	43 (28.9)	18 (30.5)	
No Confluence	33 (15.9)	21 (14.1)	12 (20.3)	
Plaque location (%)				<0.001
Right vertebral artery	64 (30.8)	48 (32.2)	16 (27.1)	
Left vertebral artery	83 (39.9)	69 (46.3)	14 (23.7)	
Basal artery	61 (29.3)	32 (21.5)	29 (49.2)	
Laboratory findings				
WBC (×10^12^/L)	6.80 [5.50, 7.91]	6.50 [5.40, 7.73]	7.20 [6.14, 8.50]	0.005
RBC (×10^9^/L)	4.44 [4.14, 4.76]	4.40 [4.12, 4.71]	4.63 [4.20, 4.89]	0.028
HGB (g/L)	136.00 [125.00, 144.93]	135.00 [125.00, 144.00]	138.00 [127.65, 148.50]	0.17
Platelets (×10^9^/L)	211.00 [169.75, 260.75]	203.00 [167.00, 256.00]	236.00 [181.00, 271.50]	0.142
NLR (%)	2.45 [1.88, 3.22]	2.36 [1.88, 3.07]	2.68 [1.91, 3.98]	0.085
PLR (%)	126.81 [100.51, 166.94]	125.27 [100.22, 169.61]	131.09 [107.95, 155.00]	0.657
HCT (%)	40.80 [37.88, 43.52]	40.60 [37.50, 43.30]	41.00 [38.20, 44.10]	0.205
MCV (fL)	91.95 [89.27, 94.53]	92.40 [89.60, 94.80]	91.20 [88.30, 93.50]	0.12
MCH (pg)	30.80 [29.78, 31.80]	31.00 [30.00, 31.90]	30.50 [29.40, 31.35]	0.051
ALT (U/L)	19.00 [12.75, 27.00]	18.00 [12.00, 26.00]	20.00 [13.00, 27.50]	0.884
AST (U/L)	19.00 [16.00, 23.00]	19.00 [16.00, 23.00]	19.00 [16.00, 23.00]	0.84
TBIL (μmol/L)	12.35 [9.70, 16.20]	12.40 [9.70, 16.00]	11.70 [9.70, 16.65]	0.511
ALB (g/L)	39.00 [36.80, 41.00]	38.80 [36.50, 41.10]	39.30 [37.20, 40.50]	0.836
Glucose (mmol/L)	5.24 [4.69, 6.80]	5.24 [4.69, 6.81]	5.24 [4.70, 6.60]	0.939
BUN (mmol/L)	5.25 [4.40, 6.52]	5.00 [4.41, 6.46]	5.76 [4.40, 6.86]	0.157
Creatinine (μmol/L)	71.30 [59.98, 84.38]	72.20 [61.20, 84.90]	67.70 [57.95, 83.40]	0.405
Uric acid (μmol/L)	324.40 [275.40, 413.98]	326.00 [276.90, 402.20]	319.60 [271.90, 426.65]	0.862
CHOL (mmol/L)	4.06 [3.36, 4.93]	3.97 [3.26, 4.73]	4.40 [3.61, 5.19]	0.037
TG (mmol/L)	1.47 [1.09, 1.95]	1.41 [1.02, 1.77]	1.60 [1.27, 2.25]	0.012
HDL (mmol/L)	1.00 [0.88, 1.13]	1.02 [0.90, 1.14]	0.94 [0.82, 1.07]	0.04
LDL (mmol/L)	2.40 [1.85, 3.02]	2.32 [1.82, 2.95]	2.72 [2.07, 3.49]	0.026
LDa (mg/L)	167.60 [75.42, 320.48]	167.70 [75.50, 336.00]	167.50 [81.70, 269.05]	0.673
HCY (umol/L)	14.20 [12.28, 16.40]	14.10 [12.40, 16.30]	14.30 [11.85, 16.80]	0.716
Fibrinogen (g/L)	328.50 [270.75, 380.00]	324.00 [267.00, 369.00]	347.00 [279.50, 392.50]	0.126
D-dimer (ng/mL)	94.00 [53.00, 164.25]	93.00 [46.00, 148.00]	107.00 [61.50, 207.50]	0.072

[ ] for IQR: interquartile range. ALB, albumin; ALT, alanine transaminase; AST, aspartate aminotransferase; BUN, blood urea nitrogen; BMI, body mass index; CHOL, total cholesterol; DBP, diastolic blood pressure; HDL, high density lipoprotein; HGB, hemoglobin; HCT, hematocrit; HCY, homocysteine; LDa, lipoprotein a; LDL, low density lipoprotein; MAP, mean arterial pressure; MCV, mean corpuscular volume; MCH, mean corpuscular hemoglobin; NLR, neutrophil-to-lymphocyte ratio; PLR, platelet-to-lymphocyte ratio; RBC, red blood cell; SBP, systolic blood pressure; TBIL, total bilirubin; TG, triglyceride; WBC, white blood cell. * Calculated with Fisher’s exact test.

## Data Availability

The data that support the findings of this study are available from the corresponding author upon reasonable request.

## Supplementary Materials

The following supporting information can be downloaded at: https://www.mdpi.com/article/10.3390/diagnostics12040812/s1, Supplementary Figure S1: (A) multivariate logistic regression of the predictors (B) Receiver operating characteristic (ROC) curves of two different predictive models. Nomogram(red) vs. Nomogram+WBC+SBP(blue)(AUC 0.83 vs. 0.845, *p* = 0.26). WBC, white blood cell; SBP, systolic blood pressure. Supplementary Figure S2: Receiver operating characteristic (ROC) curves of different predictive models using logistic regression (red), support vector machine (green) and decision tree (blue). Supplementary Figure S3: Receiver operating characteristic (ROC) curves of three different predictive models. AUC in Nomogram(red), Nom+Stenosis%(blue), Stenosis%(green) were 0.830, 0.839,0687, respectively.
