# Correction: Liu et al. A Predictive Model for the Risk of Posterior Circulation Stroke in Patients with Intracranial Atherosclerosis Based on High Resolution MRI. *Diagnostics* 2022, *12*, 812

**DOI:** 10.3390/diagnostics12092088

**Published:** 2022-08-29

**Authors:** Zhenxing Liu, Feiyang Zhong, Yu Xie, Xuanzhen Lu, Botong Hou, Keni Ouyang, Jiabin Fang, Meiyan Liao, Yumin Liu

**Affiliations:** 1 Department of Neurology, Zhongnan Hospital of Wuhan University, Wuhan 430071, China; 2 Department of Radiology, Zhongnan Hospital of Wuhan University, Wuhan 430071, China; 3 Department of Neurology, Wuhan Third Hospital, Wuhan 430060, China

Errors occurred in the number of patients in the posterior circulation ischemic stroke (PCIS) group and non-PCIS group described in the original publication [1]. A correction of the data has been made to the Abstract, Results (Section 3.1, first paragraph), Table 1 and Figure 1.

**Text Correction**

In Abstract, the sentence “They were assigned to the posterior circulation stroke (49 patients) and non-posterior circulation stroke group (159 patients) based on clinical presentation and diffusion-weighted imaging (DWI)” should be replaced with “They were assigned to the posterior circulation stroke (59 patients) and non-posterior circulation stroke group (149 patients) based on clinical presentation and diffusion-weighted imaging (DWI)”.

In Section *3.1. Clinicopathologic Characteristics of Enrolled Patients with Posterior ICAS*, the sentence “A total of 152 (73.1%) were male patients; there were 159 patients in non-posterior circulation ischemic stroke (non-PCIS) group and 49 patients in PCIS group” should be replaced with “A total of 152 (73.1%) were male patients; there were 149 patients in non-posterior circulation ischemic stroke (non-PCIS) group and 59 patients in PCIS group”.

**Table Correction**

In the first lines of Table 1, for the non-stroke group (*n* = 159) should be corrected to (*n* = 149) and for the stroke group (*n* = 49) should be corrected to (*n* = 59). The correct Table 1 is as below:

**Table 1:** Clinical and intracranial plaque characteristics of study population and comparison between stroke and non-stroke patients.

Variables	All Patients (*n* = 208)	Non-Stroke Group (*n* = 149)	Stroke Group (*n* = 59)	*p*-Value
Age (year)	61.00 [54.00, 68.00]	62.00 [54.00, 68.00]	59.00 [53.00, 66.50]	0.174
Gender				0.575
Female (%)	56 (26.9)	38 (25.5)	18 (30.5)	
Male (%)	152 (73.1)	111 (74.5)	41 (69.5)	
BMI (kg/m^2^)	25.39 [23.25, 27.14]	24.98 [23.01, 26.45]	25.95 [24.38, 27.59]	0.012
SBP (mmHg)	146.0 [131.00, 157.25]	145.00 [130.00, 153.00]	151.00 [138.50, 168.00]	0.003
DBP (mmHg)	84.00 [75.00, 95.00]	81.00 [73.00, 93.00]	87.00 [80.00, 98.00]	0.009
MAP (mmHg)	104.00 [94.67, 115.67]	102.67 [93.33, 113.33]	107.67 [100.67, 121.17]	0.003
Comorbidities, *n* (%)				
Hypertension	174 (83.7)	121 (81.2)	53 (89.8)	0.191
Diabetes	79 (38.0)	56 (37.6)	23 (39.0)	0.977
Dyslipidemia	66 (31.7)	53 (35.6)	13 (22.0)	0.084
Coronary heart disease	28 (13.5)	18 (12.1)	10 (16.9)	0.483
Previous stroke history	64 (30.8)	50 (33.6)	14 (23.7)	0.223
Smoking	92 (44.2)	67 (45.0)	25 (42.4)	0.854
Plaque characteristics				
Luminal stenosis (%)	0.41 [0.14, 0.65]	0.32 [0.10, 0.56]	0.56 [0.34, 0.88]	<0.001
Plaque burden (%)	0.80 [0.71, 0.88]	0.78 [0.69, 0.85]	0.87 [0.79, 0.96]	<0.001
Remodeling index (%)	1.10 [0.98, 1.21]	1.09 [0.97, 1.23]	1.10 [0.99, 1.17]	0.729
The type of remodeling (%)				0.655
Negative remodeling	40 (19.2)	30 (20.1)	10 (16.9)	
Intermediate remodeling	35 (16.8)	23 (15.4)	12 (20.3)	
Positive remodeling;	133 (63.9)	96 (64.4)	37 (62.7)	
Distribution patterns (%)				0.006
Diffuse	126 (60.6)	81 (54.4)	45 (76.3)	
Focal	82 (39.4)	68 (45.6)	14 (23.7)	
Quadrant Location (%)				
Ventral	151 (72.6)	103 (69.1)	48 (81.4)	0.107
Dorsal	139 (66.8)	92 (61.7)	47 (79.7)	0.021
Left	166 (79.8)	114 (76.5)	52 (88.1)	0.091
Right	144 (69.2)	96 (64.4)	48 (81.4)	0.027
Maximum wall thickness (mm)	1.46 [1.09, 2.01]	1.37 [1.02, 1.91]	1.58 [1.28, 2.11]	0.017
Maximum plaque length (mm)	5.56 [3.77, 10.57]	5.47 [3.99, 9.88]	6.45 [3.32, 11.30]	0.802
Ratio of maximum length to thickness	3.87 [2.50, 6.56]	4.17 [2.56, 6.68]	3.45 [2.26, 6.32]	0.18
Plaque enhancement (%)				<0.001 *
NO enhancement	15 (7.2)	14 (9.4)	1 (1.7)	
Mild enhancement	132 (63.5)	109 (73.2)	23 (39.0)	
Marked enhancement	61 (29.3)	26 (17.4)	35 (59.3)	
Plaque surface (%)				<0.001
Regular	73 (35.1)	65 (43.6)	8 (13.6)	
Irregular	135 (64.9)	84 (56.4)	51 (86.4)	
Geometry of the vertebrobasilar (%)				0.669
Walking	55 (26.4)	41 (27.5)	14 (23.7)	
Tuning Fork	59 (28.4)	44 (29.5)	15 (25.4)	
Lambda	61 (29.3)	43 (28.9)	18 (30.5)	
No Confluence	33 (15.9)	21 (14.1)	12 (20.3)	
Plaque location (%)				<0.001
Right vertebral artery	64 (30.8)	48 (32.2)	16 (27.1)	
Left vertebral artery	83 (39.9)	69 (46.3)	14 (23.7)	
Basal artery	61 (29.3)	32 (21.5)	29 (49.2)	
Laboratory findings				
WBC (×10^12^/L)	6.80 [5.50, 7.91]	6.50 [5.40, 7.73]	7.20 [6.14, 8.50]	0.005
RBC (×10^9^/L)	4.44 [4.14, 4.76]	4.40 [4.12, 4.71]	4.63 [4.20, 4.89]	0.028
HGB (g/L)	136.00 [125.00, 144.93]	135.00 [125.00, 144.00]	138.00 [127.65, 148.50]	0.17
Platelets (×10^9^/L)	211.00 [169.75, 260.75]	203.00 [167.00, 256.00]	236.00 [181.00, 271.50]	0.142
NLR (%)	2.45 [1.88, 3.22]	2.36 [1.88, 3.07]	2.68 [1.91, 3.98]	0.085
PLR (%)	126.81 [100.51, 166.94]	125.27 [100.22, 169.61]	131.09 [107.95, 155.00]	0.657
HCT (%)	40.80 [37.88, 43.52]	40.60 [37.50, 43.30]	41.00 [38.20, 44.10]	0.205
MCV (fL)	91.95 [89.27, 94.53]	92.40 [89.60, 94.80]	91.20 [88.30, 93.50]	0.12
MCH (pg)	30.80 [29.78, 31.80]	31.00 [30.00, 31.90]	30.50 [29.40, 31.35]	0.051
ALT (U/L)	19.00 [12.75, 27.00]	18.00 [12.00, 26.00]	20.00 [13.00, 27.50]	0.884
AST (U/L)	19.00 [16.00, 23.00]	19.00 [16.00, 23.00]	19.00 [16.00, 23.00]	0.84
TBIL (μmol/L)	12.35 [9.70, 16.20]	12.40 [9.70, 16.00]	11.70 [9.70, 16.65]	0.511
ALB (g/L)	39.00 [36.80, 41.00]	38.80 [36.50, 41.10]	39.30 [37.20, 40.50]	0.836
Glucose (mmol/L)	5.24 [4.69, 6.80]	5.24 [4.69, 6.81]	5.24 [4.70, 6.60]	0.939
BUN (mmol/L)	5.25 [4.40, 6.52]	5.00 [4.41, 6.46]	5.76 [4.40, 6.86]	0.157
Creatinine (μmol/L)	71.30 [59.98, 84.38]	72.20 [61.20, 84.90]	67.70 [57.95, 83.40]	0.405
Uric acid (μmol/L)	324.40 [275.40, 413.98]	326.00 [276.90, 402.20]	319.60 [271.90, 426.65]	0.862
CHOL (mmol/L)	4.06 [3.36, 4.93]	3.97 [3.26, 4.73]	4.40 [3.61, 5.19]	0.037
TG (mmol/L)	1.47 [1.09, 1.95]	1.41 [1.02, 1.77]	1.60 [1.27, 2.25]	0.012
HDL (mmol/L)	1.00 [0.88, 1.13]	1.02 [0.90, 1.14]	0.94 [0.82, 1.07]	0.04
LDL (mmol/L)	2.40 [1.85, 3.02]	2.32 [1.82, 2.95]	2.72 [2.07, 3.49]	0.026
LDa (mg/L)	167.60 [75.42, 320.48]	167.70 [75.50, 336.00]	167.50 [81.70, 269.05]	0.673
HCY (umol/L)	14.20 [12.28, 16.40]	14.10 [12.40, 16.30]	14.30 [11.85, 16.80]	0.716
Fibrinogen (g/L)	328.50 [270.75, 380.00]	324.00 [267.00, 369.00]	347.00 [279.50, 392.50]	0.126
D-dimer (ng/mL)	94.00 [53.00, 164.25]	93.00 [46.00, 148.00]	107.00 [61.50, 207.50]	0.072

[ ] for IQR: interquartile range. ALB, albumin; ALT, alanine transaminase; AST, aspartate aminotransferase; BUN, blood urea nitrogen; BMI, body mass index; CHOL, total cholesterol; DBP, diastolic blood pressure; HDL, high density lipoprotein; HGB, hemoglobin; HCT, hematocrit; HCY, homocysteine; LDa, lipoprotein a; LDL, low density lipoprotein; MAP, mean arterial pressure; MCV, mean corpuscular volume; MCH, mean corpuscular hemoglobin; NLR, neutrophil-to-lymphocyte ratio; PLR, platelet-to-lymphocyte ratio; RBC, red blood cell; SBP, systolic blood pressure; TBIL, total bilirubin; TG, triglyceride; WBC, white blood cell. * Calculated with Fisher’s exact test.

**Figure Correction**

In the last lines of Figure 1, for the group of the patiens with non-posterior circulation ischemic stroke, (*n* = 159) should be corrected to (*n* = 149), and for the group of patiens with posterior circulation ischemic stroke, (*n* = 49) should be corrected to (*n* = 59). The correct Figure 1 is as below:

**Figure 1:**
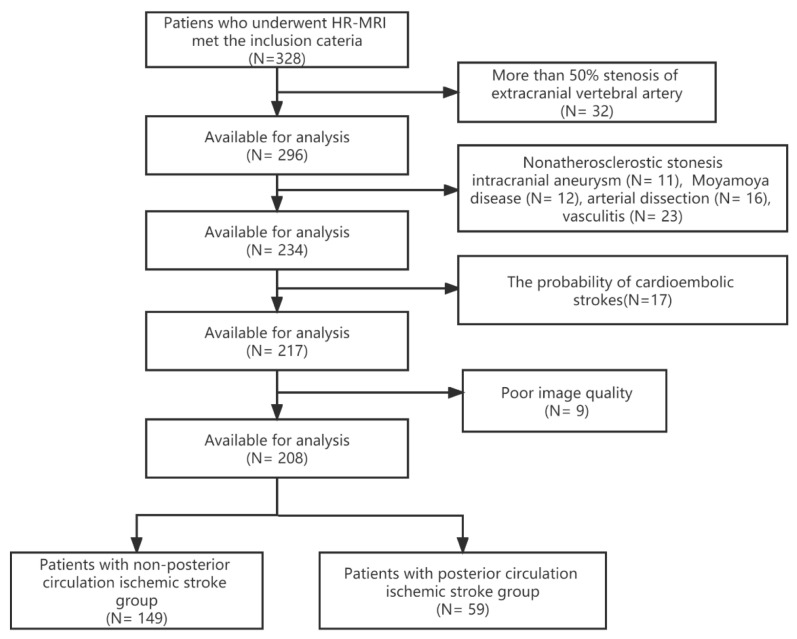
The flow chart for the inclusion of patients. HR-MRI, high-resolution magnetic resonance imaging.

The authors apologize for any inconvenience caused and state that the scientific conclusions are unaffected. This correction was approved by the Academic Editor. The original publication has also been updated.

## References

[1] Liu Z., Zhong F., Xie Y., Lu X., Hou B., Ouyang K., Fang J., Liao M., Liu Y. (2022). A predictive model for the risk of posterior circulation stroke in patients with intracranial atherosclerosis based on high resolution MRI. Diagnostics.

