# Supplementary material for: A Predictive Model for the Risk of Posterior Circulation Stroke in Patients with Intracranial Atherosclerosis Based on High Resolution MRI
Source: Diagnostics (Basel). 2022 Mar 15;12(4):812. doi: 10.3390/diagnostics12040812 (PMC9031625; doi:10.3390/diagnostics12040812)
Supplement: Supplementary file 1 [file diagnostics-12-00812-s001.zip › diagnostics-1568678-supplementary.pdf]

Supplementary Materials:

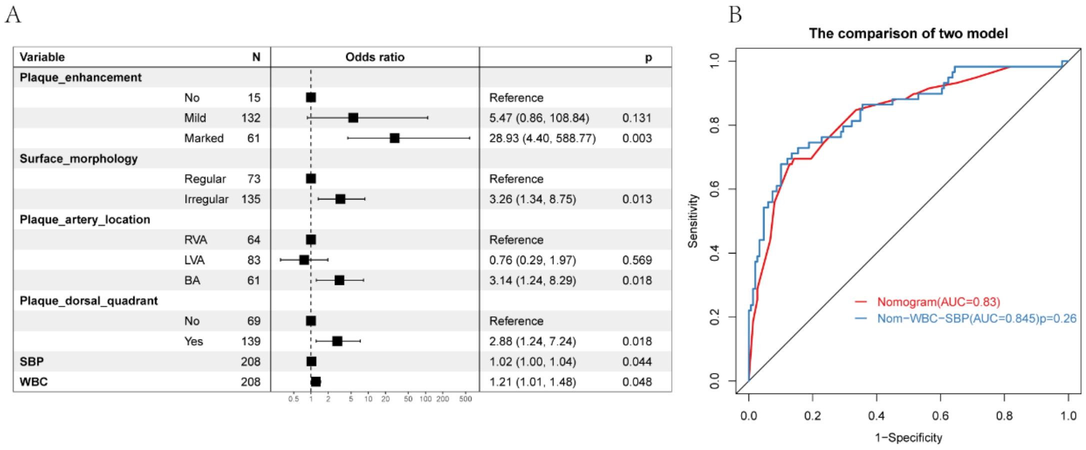

Supplementary Figure S1: (**A**) multivariate logistic regression of the predictors (**B**) Receiver operating characteristic (ROC) curves of two different predictive models. Nomogram(red) vs. Nomogram+WBC+SBP(blue)(AUC 0.83 vs. 0.845, p=0.26). WBC, white blood cell; SBP, systolic blood pressure

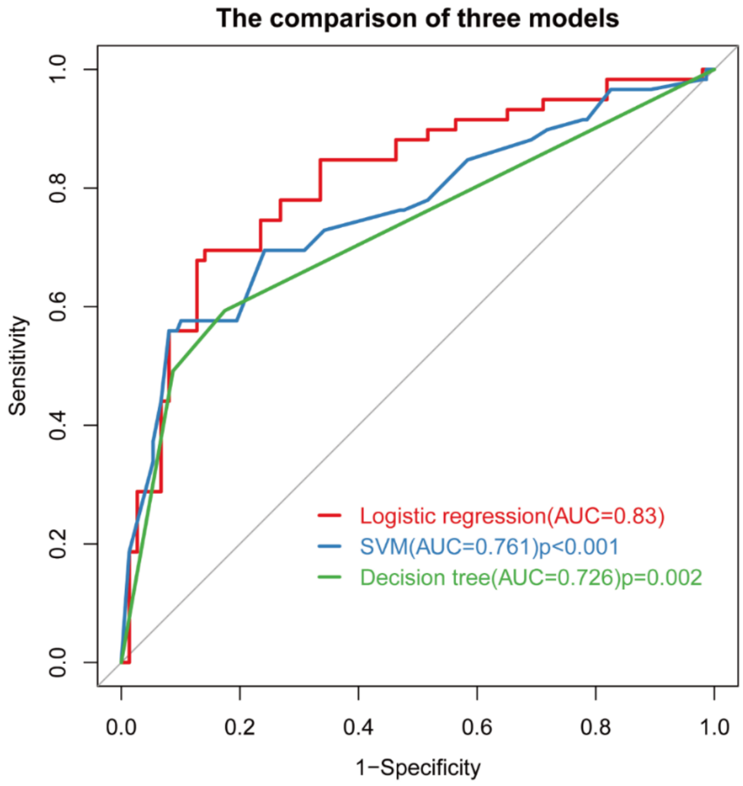

Supplementary Figure S2: Receiver operating characteristic (ROC) curves of different predictive models using logistic regression (red), support vector machine (green) and decision tree (blue).

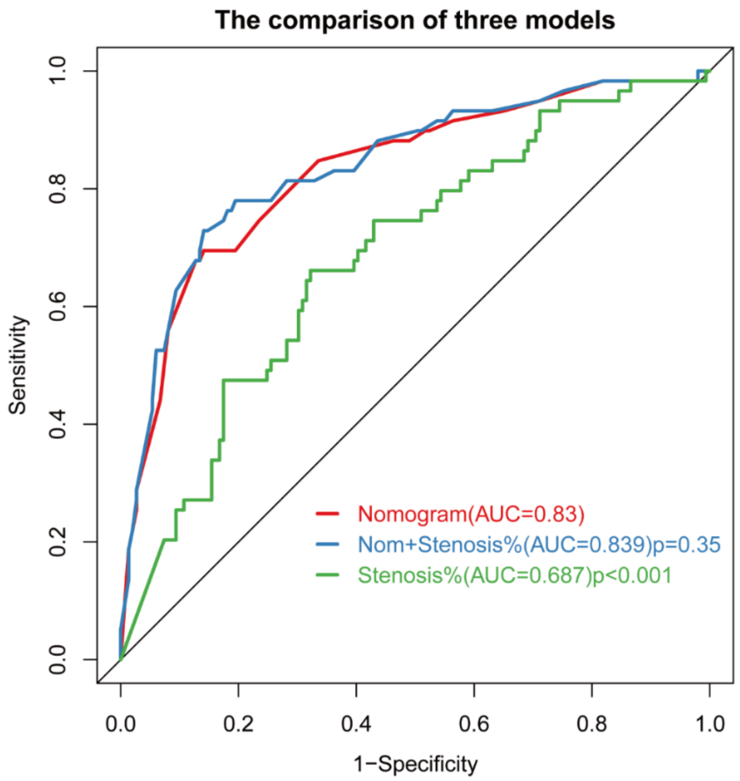

Supplementary Figure S3: Receiver operating characteristic (ROC) curves of three different predictive models. AUC in Nomogram(red), Nom+Stenosis%(blue), Stenosis%(green) were 0.830, 0.839,0687, respectively.
